# Customized online and onsite training for rabies-control officers 

**DOI:** 10.2471/BLT.14.149849

**Published:** 2015-04-27

**Authors:** Hervé Bourhy, Cécile Troupin, Ousmane Faye, François-Xavier Meslin, Bernadette Abela-Ridder, Amadou Alpha Sall, Jean-Pierre Kraehenbuhl

**Affiliations:** a Institut Pasteur, National Reference Centre for Rabies, Paris, France.; b Institut Pasteur, Dakar, Senegal.; c Grand Saconnex, Switzerland.; d Department for the Control of Neglected Tropical Diseases, World Health Organization, Geneva, Switzerland.; e Health Sciences eTraining (HSeT) Foundation, 155 Chemin des Boveresses, 1066 Epalinges, Switzerland.

## Abstract

**Problem:**

It is difficult to deliver adequate training for people working in rabies control in low and middle-income countries. Popular e-learning systems for low-income settings are not well suited to developing and testing practical skills, including laboratory methods.

**Approach:**

We customized training in rabies control methods for African professionals and students from different disciplines. Trainees participated in preparatory online sessions, evaluations and exercises before and after a 12-day workshop. Trainees and mentors continued to interact through an online forum up to one year after the workshop.

**Local setting:**

In Africa, 15 000 deaths from rabies occur each year due to a lack of awareness, inaccessibility of post-exposure prophylaxis, inadequate or absent canine rabies-control programmes and lack of governmental financial support.

**Relevant changes:**

Thirty two trainees – working in health departments, hospitals, veterinary stations and research institutes – were selected to participate; 28 completed the course and passed the final evaluation. Pilot rabies investigation programmes were developed, and two manuscripts submitted for publication. An online forum facilitated further progress for a year after the workshop.

**Lessons learnt:**

A combination of customized online and onsite training is suitable for teaching disease-control personnel in low-income countries. Participation in this course enabled trainees to advocate for the development of national disease-control strategies. Mentoring is needed to develop a strong network of experts in similar settings.

## Résumé

**Problème:**

Il est difficile de dispenser une formation appropriée aux personnes intervenant dans la lutte contre la rage dans les pays à revenu faible et intermédiaire. Les systèmes populaires d'apprentissage en ligne conçus pour les pays à faible revenu ne sont pas adaptés au développement et au test des compétences pratiques, notamment en ce qui concerne les méthodes analytiques.

**Approche:**

Nous avons adapté la formation sur les méthodes de lutte contre la rage à l'intention de professionnels et d'étudiants africains issus de différentes disciplines. Les bénéficiaires de la formation ont participé à des séances de préparation en ligne, à des évaluations et à des exercices, avant et après un atelier de 12 jours. Puis, les bénéficiaires de la formation et les tuteurs ont continué à interagir jusqu'à un an après l'atelier via un forum accessible en ligne.

**Environnement local:**

En Afrique, 15.000 décès dus à la rage surviennent chaque année. Cette situation vient d'un manque de sensibilisation, de l'inaccessibilité des traitements de prophylaxie post-exposition, de programmes de lutte contre la rage canine inappropriés voire inexistants et d'un manque de soutien financier gouvernemental.

**Changements significatifs:**

Trente-deux bénéficiaires de la formation (qui travaillent dans des services de santé, des hôpitaux, des centres vétérinaires ou des instituts de recherche) ont été sélectionnés pour la formation ; vingt-huit l'ont achevée et ont passé avec succès l'évaluation finale. Des programmes pilotes d'enquête sur la rage ont été élaborés et deux manuscrits ont été soumis pour publication. Un forum en ligne a facilité la poursuite des progrès pendant l'année qui a suivi l'atelier.

**Leçons tirées:**

L'association de modules proposés en ligne et sur place est adaptée à la formation du personnel de lutte contre la maladie dans les pays à faible revenu. La participation à cette formation a permis à ses bénéficiaires de plaider pour l'élaboration de stratégies nationales de lutte contre la maladie. Un tutorat est nécessaire pour constituer un solide réseau d'experts dans des contextes similaires.

## Resumen

**Situación:**

Es difícil proporcionar una formación adecuada a aquellos que trabajan en el control de la rabia en países de ingresos bajos y medios. Los populares sistemas de aprendizaje en línea en comunidades de ingresos bajos no son adecuados para desarrollar y poner a prueba las habilidades prácticas, incluidos los métodos de laboratorio.

**Enfoque:**

Se personalizó la formación en los métodos de control de la rabia para los profesionales y estudiantes africanos de diferentes disciplinas. Los alumnos participaron en sesiones preparatorias, evaluaciones y ejercicios en línea antes y después de un taller de 12 días. Los alumnos y los profesores siguieron interactuando a través de un foro en línea hasta un año después del taller.

**Marco regional:**

En África, hay 15.000 muertes de rabia cada año debidas a la falta de concienciación, la inaccesibilidad de la profilaxis postexposición, los programas inadecuados o ausentes de control de la rabia canina y la falta de apoyo financiero del gobierno.

**Cambios importantes:**

Treinta y dos alumnos que trabajan en departamentos de salud, hospitales, clínicas veterinarias e institutos de investigación fueron seleccionados para participar. 28 de ellos completaron el curso y aprobaron la evaluación final. Se desarrollaron programas piloto de investigación de la rabia y se presentaron 2 manuscritos para su publicación. Gracias al foro en línea, se pudo seguir progresando hasta un año después del taller.

**Lecciones aprendidas:**

La combinación de la formación en línea y presencial es adecuada para enseñar al personal de control de enfermedades en países de ingresos bajos. La participación en este curso permitió a los alumnos promocionar el desarrollo de estrategias nacionales de control de enfermedades. La tutoría es necesaria para desarrollar una sólida red de expertos en marcos similares.

## ملخص

**المشكلة:**

يصعب تقديم التدريب الكافي للعاملين في مكافحة داء الكَلَب في البلدان منخفضة ومتوسطة الدخل. ولا تُعد نظم التعليم الإلكتروني الشعبية للمواقع منخفضة الدخل مناسبة تمامًا لتطوير واختبار المهارات العملية، بما في ذلك الأساليب المختبرية.

**الأسلوب:**

لقد قمنا بتخصيص تدريبات في أساليب مكافحة داء الكَلَب للمختصين الأفارقة والطلبة من مختلف الاختصاصات. وشارك المتدربون في الجلسات والتقييمات والتمارين التحضيرية المباشرة على شبكة الإنترنت، قبل وبعد إجراء حلقة عمل لمدة **  **12 **  **يومًا. واستمر المتدربون والموجهون في التفاعل من خلال منتدى مباشر على شبكة الإنترنت لمدة تصل إلى عام واحد بعد إجراء حلقة العمل.

**المواقع المحلية:**

في أفريقيا، تحدث **  **15,000 **  **حالة وفاة جراء الإصابة بداء الكَلَب كل عام بسبب نقص الوعي، وصعوبة الحصول على العلاج الوقائي بعد التعرض للفيروس، وعدم كفاية أو غياب برامج مكافحة داء الكَلَب، ونقص الدعم المالي الحكومي

**التغيّرات ذات الصلة:**

تم اختيار اثنان وثلاثين متدربًا – يعملون في الأقسام والإدارات الصحية، والمستشفيات، والمحطات البيطرية، ومعاهد الأبحاث – للمشاركة؛ وقد قام **  **28 **  **متدربًا منهم بإتمام الدورة واجتياز التقييم النهائي. لقد تم إعداد برامج تجريبية لاستقصاء داء الكَلَب، وتم إرسال مخطوطتين للنشر. وقام منتدى مباشر على شبكة الإنترنت بتيسير إحراز المزيد من التقدم لمدة عام بعد إجراء حلقة العمل.

**الدروس المستفادة:**

من المناسب الجمع بين التدريبات المباشرة على الإنترنت والتدريبات الميدانية في الموقع لتعليم العاملين المختصين بمكافحة الأمراض في البلدان منخفضة الدخل. كان للمشاركة في هذه الدورة التدريبية أثر في تمكين المتدربين من الدعوة إلى إعداد استراتيجيات وطنية لمكافحة الأمراض. وهناك حاجة إلى التوجيه لإعداد شبكة قوية من الخبراء في بيئات مشابهة.

## 摘要

**问题:**

在中低收入国家中难以为从事狂犬病控制的人员提供合适的培训。低收入环境中普及的电子学习系统不太适于开发并测试实践技能，包括化验方法。

**方法:**

我们针对不同学科的非洲专业人员和学生开展了关于狂犬病控制方法的定制化培训。受训人员在为期 12 天的研讨会前后参与了预备在线课程、评估和练习。受训人员和导师在研讨会结束后继续通过在线论坛进行了长达一年的互动交流。

**当地状况:**

在非洲，每年有 15 000 人死于狂犬病，这是因为缺乏意识、无法在接触后采取预防措施、狂犬病控制计划的制定不充分或没有制定，以及政府财政支持不足。

**相关变化:**

选出 32 名就职于卫生部门、医院、兽医站和研究所的受训人员参与培训，其中有 28 名完成了课程并通过了最终评估。制定出试行的狂犬病调查计划，并提交了 2 份原稿用于出版。研讨会结束后，在线论坛进一步推动了为期一年的进展。

**经验教训:**

定制化的在线和现场培训相结合，适合用于教导低收入国家的疾病控制人员。参与此项课程有助于让受训人员拥护国家疾病控制策略的发展。在相似的环境中，需要在指导下开发强大的专家网络。

## Резюме

**Проблема:**

В странах с низким и средним уровнем доходов населения трудно обеспечить адекватное обучение специалистов по контролю распространения бешенства. Популярные системы электронного обучения для стран с низким уровнем дохода плохо подходят для развития и проверки практических навыков, включая знания лабораторных методов контроля.

**Подход:**

Обучение методам контроля распространения бешенства было адаптировано для африканских специалистов и студентов различных дисциплин. Обучаемые проходили 12-дневный практический курс. Перед курсом и после него проводились подготовительные занятия, тестирование и практические занятия в режиме онлайн. Обучаемые и кураторы продолжали взаимодействие через онлайн-форум на протяжении одного года после окончания курса.

**Местные условия:**

В Африке от бешенства ежегодно умирает 15 000 человек. Причиной тому является слабая информированность населения, недоступность постконтактной профилактики, неэффективность или отсутствие программ контроля распространения бешенства среди собак и отсутствие финансовой поддержки от государства.

**Осуществленные перемены:**

Для участия в курсе были отобраны 32 сотрудника из департаментов здравоохранения, больниц, ветеринарных станций и исследовательских институтов; 28 человек окончили курс и прошли окончательное тестирование. Были разработаны пилотные программы по исследованию бешенства, и две рукописи были сданы в печать. Онлайн-форум способствовал дальнейшему обучению участников на протяжении одного года после окончания курса.

**Выводы:**

Для обучения специалистов по контролю распространения заболеваний в странах с низким уровнем дохода населения подходит сочетание индивидуализированного обучения онлайн и на местах. Участие в данном курсе позволило обучаемым выступить в поддержку разработки национальных стратегий по контролю распространения заболеваний. Для создания сети высококвалифицированных специалистов в подобных условиях необходимо организовать эффективный процесс наставничества.****

## Problem

In low- and middle-income countries, it can be difficult to deliver adequate training for people working in disease control. Many e-training programmes are based on participatory learning models in which participants share their understanding and monitor their theoretical knowledge through discussion, questioning and interaction with mentors via the internet. The current most popular e-learning systems for resource-poor settings are massive online open courses^1^^,^^2^ which have been used by tens of thousands of students around the globe.^3^ However, this format is not well suited for specific practical training needs.

Scientists and public-health professionals working in neighbouring countries often miss opportunities to exchange information because collaborations have been traditionally formed between distant, high-income countries. Public health managers and scientists are often trained separately, making it difficult to link human and animal health at national and international levels.

Our aim was to provide practical training on rabies prevention in Africa for students and professionals in animal and human public health sectors.^4^^,^^5^ We also wanted to address critical gaps in rabies control as have been described elsewhere.^6^^–^^8^

## Approach

We used an approach called customized online training, (COLT) which focuses on small sets of trainees and is designed for situations where acquisition of skills and direct training by experts are needed. With this approach, it is feasible to tailor training to each individual trainee in a way that would be impractical in a system designed for mass audiences. The COLT approach has been used successfully for several workshops and courses in Africa, Asia and South America (http://octave.bio-med.ch). We describe here the use of the COLT approach in a course on the control and surveillance of rabies organized in Dakar, Senegal, in December 2013. The workshop was organized by the Pasteur Institutes in Dakar and Paris, the Health Sciences eTraining Foundation and the World Health Organization (WHO). It was held in the *Ecole Inter-Etats des Sciences et Médecine Vétérinaire* in Dakar over a period of nine days. Practical training activities were given at the Institut Pasteur of Dakar, at the Fann Hospital in Dakar and during three days in the municipality of M'bour, south of Dakar. Training in M’bour included knowledge, attitude and practice surveys, dog-population surveys and dog-vaccination campaigns.

## Local setting

Rabies is a lethal encephalitis due to a lyssavirus mainly transmitted to humans by the bite or scratches of infected animals. Approximately 15 000 deaths from rabies occur in Africa each year due to a lack of awareness about the disease and the consequences of dog bites, lack of access to post-exposure prophylaxis, and inadequate or absent canine rabies-control programmes.

## Relevant changes

We designed a workshop for physicians, veterinarians, public health officers and specialists in infectious diseases, virology and/or epidemiology. Participants needed to be fluent in French and have at least a bachelor degree and preferably a master’s degree. The course was advertised through a website and by participating international organizations and regional networks. Most of the 106 applicants were from Francophone African countries. Trainees were selected on the basis of a curriculum vitae, letter of motivation and three letters of recommendation. Thirty-two participants working in national and regional veterinary stations, hospitals or research institutes in 13 countries (Algeria, Burkina Faso, Cameroon, Chad, Congo, Côte d’Ivoire, Democratic Republic of the Congo, Madagascar, Mali, Niger, Senegal, South Africa, Togo) were selected and encouraged to start pre-workshop activities.

### Online pre-workshop activities

An online pre-training assessment tool was sent to participants, who completed this before starting a set of three activities. First, trainees were provided with annotated articles on rabies and asked to respond to questions on epidemiology, pathogenesis, laboratory methods of diagnosis, statistics and clinical features of infection. Modules could be downloaded to allow the trainees to study despite irregular internet access. Second, as a virtual team exercise, trainees were asked to write a national plan for the control and surveillance of rabies in Senegal. The trainees had to interact with each other using an online forum and address specific questions that were provided by the experts. Third, trainees had to write a manuscript on their research related to rabies, guided by an application developed by Jonathan Fuchs (Department of Public Health, San Francisco, United States of America). These activities required about 70 hours of individual work, followed by a second online evaluation.

### Workshop activities

The onsite workshop, over 12 days, focused on practical sessions including: testing rabid dog-brain samples by immunofluorescence or by reverse transcriptase polymerase chain reaction (RT–PCR); anti-rabies antibody detection; analysing ribonucleic acid (RNA) sequences; and, estimating the growth rate of epidemics. The 24 teachers came from seven countries: Cambodia (1), France (4), Italy (1), Senegal (12), South Africa (1), Switzerland (3) and the United Kingdom of Great Britain and Northern Ireland (2).

The organization of the workshop activities favoured debates, discussions and analysis of local contingencies to find practical, economical and reliable solutions to the current rabies situation in Senegal. Work done during the pre-workshop period was finished during the workshop and presented to the panel of experts. The experts made recommendations and forwarded the proposed national plan, including a budget, to the ministries in charge of rabies control and surveillance in Senegal.

To assess the effectiveness of the workshop process, we held a final examination. Twenty-eight trainees succeeded with scores above 74% (range: 74–88%). The trainees attending the whole course received five credits from the European credit transfer and accumulation system, delivered by Lausanne University, Switzerland. The completion rate of the course (86%; 28/32) was higher than for many massive open online courses, which often have completion rates below 20%.^9^ All trainees completed a final evaluation in which they provided feedback on the course and indicated how they planned to transfer the knowledge and skills they had acquired to their daily practice.

### Post-workshop activities

During the year following the course, discussions on the online forum continued between the participants and the teachers. Pilot rabies investigation programmes were developed by the participants and sent to the experts for review and then submitted to national or international funding agencies. The participants organized other courses on rabies control and prevention. These were held at the local or provincial level and in Côte d'Ivoire, Madagascar and South Africa, at the national level. Eight manuscripts on rabies-related topics were written and two have been accepted for publication in international peer-reviewed journals.

These outcomes illustrate the intensity of the post-workshop activities, the need for an ongoing training component and the value of mentoring provided before, during and after the workshop. Mentoring is a core component of medical education and career success and should be promoted in low- and middle-income countries.^10^ Box 1 summarizes the main lessons learnt during this workshop.

Box 1 **Summary of main lessons learnt**

- Customized online training is suitable for disease-control programmes in low-income countries.
- Online pre-workshop activities are essential for preparing participants for the workshop.
- Mentoring is needed to create a strong network of disease-control experts working in similar settings.

### Costs and human resources

The budget for this course was 3000 Euros per trainee. This covered creating and maintaining the website, managing the online forum, travel to Senegal and local expenses for trainees and experts. The participants paid no registration fees. Of the 24 teachers present during the workshop, 14 were experts working on rabies in ministries and research institutions in Senegal. This mix of foreign and local teachers helped to ensure the support of the host country and meant that debates between participants were informed by a very good understanding of the local situation in the field.
